# Supplementary material for: Using statistical modelling and machine learning in detecting bone properties: A systematic review protocol
Source: PLoS One. 2025 Mar 11;20(3):e0319583. doi: 10.1371/journal.pone.0319583 (PMC11896029; doi:10.1371/journal.pone.0319583)
Supplement: S2 File — (DOCX) [file pone.0319583.s002.docx]

An example of the search strategy for PubMed is as follows:

("Artificial Intelligence"[Mesh] OR "Artificial Intelligence" OR "Machine Learning"[Mesh] OR "Machine Learning" OR "Deep Learning" OR "Neural Networks (Computer)" OR "Neural Networks" OR "Decision Trees" OR "Support Vector Machine" OR "Random Forest" OR "Supervised Learning" OR "Unsupervised Learning" OR "Reinforcement Learning" OR "Natural Language Processing")

AND

("Osteoporosis"[Mesh] OR "Osteoporosis" OR "Bone Density"[Mesh] OR "Bone Density" OR "Bone Mineral Density" OR "BMD" OR "Bone Health" OR "Bone Strength" OR "Bone Properties" OR "Bone Microarchitecture" OR "Vertebral Fracture" OR "Fracture Risk" OR "Bone Loss")

AND

("Diagnosis"[Mesh] OR "Diagnosis" OR "Detection" OR "Prediction" OR "Assessment" OR "Screening" OR "Risk Assessment")

AND

("Adults" OR "Older Adults" OR "Elderly" OR "Postmenopausal Women" OR "Middle Aged")

NOT

("Animals"[Mesh] OR "Animal Experimentation" OR "Animal Studies" OR "Non-Human")
