# Supplementary material for: Using statistical modelling and machine learning in detecting bone properties: A systematic review protocol
Source: PLoS One. 2025 Mar 11;20(3):e0319583. doi: 10.1371/journal.pone.0319583 (PMC11896029; doi:10.1371/journal.pone.0319583)
Supplement: Attachment — Submitted filename: Response letter to the reviewers.docx [file pone.0319583.s003.docx]

**Response letter to the reviewers**

**Reviewer #2 Comments**

**Comment 1**

*“The manuscript tackles a critical and timely issue by exploring the application of AI and ML in detecting and predicting osteoporosis-related bone properties. This area of research is highly relevant, as traditional diagnostic methods like DXA face limitations in sensitivity and accessibility, leaving room for innovative technologies to make an impact.”*

**Response:**

- We thank the reviewer for this comment.

**Comment 2**

*“The authors have presented a strong and methodologically rigorous protocol that adheres to PRISMA-P guidelines. Their use of established risk-of-bias tools, such as RoB 2 and QUADAS-2, ensures the reliability and transparency of the review. This approach enhances the credibility of the proposed study.”*

**Response:**

- We thank the reviewer for this comment

**Comment 3**

*“A notable strength is the interdisciplinary nature of the protocol, which incorporates a variety of AI/ML methods and data types, including imaging, genomic data, and wearable sensor data. This comprehensive scope allows for a broad understanding of AI/ML applications in osteoporosis detection and prediction.”*

**Proposed Revision/Response:**

- We thank the reviewer for this comment

**Comment 4**

*“However, the decision to include only English-language studies introduces potential language bias. Considering the global nature of osteoporosis research, this could exclude significant findings published in other languages. Addressing this limitation or providing a clear rationale for this choice would strengthen the manuscript.”*

**Proposed Revision/Response:**

1. **Rationale Explanation**: We have added a subsection under “Search Strategy”, explaining that we have limited the search to English-language studies due to resource constraints (e.g., translation resources, reviewer expertise).
2. **Acknowledgment of Limitation**: In the “Discussion” or “Limitations” section, we have emphasised that future reviews could broaden the language scope.

**Comment 5**

*“While the authors acknowledge the challenge of rapidly evolving AI/ML technologies, the protocol does not outline a concrete strategy for updating the review with newly published studies. Including a plan to address this would ensure the review remains relevant and comprehensive over time.”*

**Proposed Revision/Response:**

- We have added a section under the methodology about performing a “living systematic review” approach or scheduling periodic updates (i.e., every 12 months) to capture relevant new AI/ML methods in osteoporosis research.
- We have also noted that automated alerts from databases (e.g., PubMed’s My NCBI) will be set up to track newly published relevant literature.

Elliott JH, Synnot A, Turner T, et al. “Living systematic review: An emerging opportunity to narrow the evidence–practice gap.” *PLoS Medicine*, 14(1), e1002280, 2017. https://doi.org/10.1371/journal.pmed.1002280

**Comment 6**

*“The absence of illustrative examples or pilot data is a missed opportunity to provide readers with practical context. Even a hypothetical case or brief summary of existing AI/ML applications in osteoporosis could help clarify the practical implications of the study.”*

**Proposed Revision/Response:**

- We incorporated a concise subsection briefly describing an example use case (hypothetical based on the literature) demonstrating how AI/ML might identify subtle bone mineral density changes from hip X-rays.

**Comment 7**

*“The discussion section could be expanded to better address the real-world impact of the review. For example, how could these findings influence clinical workflows, public health strategies, or the development of guidelines for integrating AI/ML into osteoporosis care?”*

**Proposed Revision/Response:**

- We expanded the “Discussion” section to include specific points on how clinicians could implement AI/ML tools in routine workflows (e.g., automated BMD measurements in radiology reports and integration with electronic health records).
- We discussed broader public health implications, including how predictive modelling might guide population screening strategies or resource allocation in ageing populations.
- We added a subsection about the future directions and the impact of systematic reviews such as ours on establishing evidence-based practices.

**Comment 8**

*“While the manuscript is generally well-written and logically structured, some technical terms (e.g., ensemble methods, reinforcement learning) could be simplified or briefly explained to ensure accessibility for a broader audience, including clinicians and policymakers.”*

**Proposed Revision/Response:**

- We added short footnotes to explain key AI/ML technical terms (e.g., ensemble methods, reinforcement learning, deep learning) in non-technical language.

**Comment 9**

*“The authors’ commitment to making all data publicly available upon study completion aligns well with open science principles and enhances the transparency of the work. This is a commendable practice.”*

**Proposed Revision/Response:**

- We thank the reviewer for this comment

**Comment 10**

*“Overall, this protocol is well-conceived and has the potential to contribute significantly to the field. By addressing limitations such as language bias, providing practical examples, and elaborating on the broader implications of the findings, the manuscript could be further strengthened and have a more impactful reach.”*

**Proposed Revision/Response:**

- We thank the reviewer for this comment. We believe the comments we received have enhanced our protocol. We hope the modifications the manuscript underwent align with the reviewer's comment.

**Reviewer #3 Comments**

**Comment**

*“Your proposal for SR and MA about this issue is meaningful and necessary; I would recommend, however, to narrow your inclusion criteria for adults over 60 years old; this recommendation, although it may limit the amount of studies available for review, it also may produce more meaningful results and avoid bias from analysing very different populations in the same MA. Another solution for this would be to analyse younger adults (< 60 YO) separately from older adults.”*

**Proposed Revision/Response:**

- We thank the reviewer for this comment. To reduce the bias concern that the reviewer mentioned, we mentioned in the methodology that sensitivity and stratified analysis would be considered to account for potential sources of bias and heterogeneity. Moreover, given enough power and sample size, i.e., the number of studies and subjects within the studies, we aim to produce a meta-regression to reduce confounding effects.
